# Methyl 2,2-dimeth­oxy-5,5-bis­(methyl­sulfan­yl)-3-oxopent-4-enedithio­ate

**DOI:** 10.1107/S1600536812011282

**Published:** 2012-03-21

**Authors:** Mohammad Hassan Ghorbani

**Affiliations:** a Falavarjan Branch, Islamic Azad University, Falavarjan, Isfahan, Iran

## Abstract

In the title mol­ecule, C_10_H_16_O_3_S_4_, a short intra­molecular S⋯O(=C) distance [2.726 (2) Å] indicates the presence of a nonbonding attractive inter­action. In the crystal, mol­ecules are linked into centrosymmetric dimers *via* weak inter­molecular C—H⋯O and S⋯S [3.405 (3) Å] inter­actions. These dimers are linked by further weak C—H⋯O inter­actions into columns along the *a* axis.

## Related literature

For background and synthetic details, see: Mahata *et al.* (2003 ▶). For related structures and S⋯O interactions, see: Ángyán *et al.* (1985 ▶, 1987 ▶); Dixit *et al.* (1995 ▶); Hamilton & LaPlaca (1964 ▶). For van der Waals radii, see: Ángyán *et al.* (1987 ▶). For S⋯S inter­actions, see: Guru Row & Parthasarathy (1981 ▶); Puranik *et al.* (1986 ▶). 
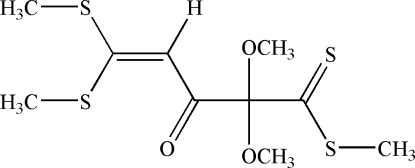


## Experimental

### 

#### Crystal data

C_10_H_16_O_3_S_4_
*M* *_r_* = 312.47
Triclinic, 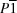

*a* = 7.114 (5) Å
*b* = 10.404 (5) Å
*c* = 11.151 (5) Å
α = 70.426 (5)°
β = 88.549 (5)°
γ = 76.081 (5)°
*V* = 753.4 (7) Å^3^
*Z* = 2
Mo *K*α radiation
μ = 0.62 mm^−1^
*T* = 291 K
0.35 × 0.25 × 0.12 mm

#### Data collection

Stoe IPDS II Image Plate diffractometer
Absorption correction: multi-scan [*MULABS* (Blessing, 1995 ▶) in *PLATON* (Spek, 2009 ▶)] *T*_min_ = 0.927, *T*_max_ = 1.000
8030 measured reflections
4016 independent reflections
2376 reflections with *I* > 2σ(*I*)
*R*_int_ = 0.036

#### Refinement

*R*[*F* ^2^ > 2σ(*F* ^2^)] = 0.037
*wR*(*F* ^2^) = 0.085
*S* = 0.81
4016 reflections
160 parameters
H-atom parameters constrained
Δρ_max_ = 0.29 e Å^−3^
Δρ_min_ = −0.35 e Å^−3^

### 

Data collection: *X-AREA* (Stoe & Cie, 2005 ▶); cell refinement: *X-AREA*; data reduction: *X-AREA*; program(s) used to solve structure: *SHELXTL* (Sheldrick, 2008 ▶); program(s) used to refine structure: *SHELXTL*; molecular graphics: *SHELXTL*; software used to prepare material for publication: *SHELXTL* and *PLATON* (Spek, 2009 ▶).

## Supplementary Material

Crystal structure: contains datablock(s) I, global. DOI: 10.1107/S1600536812011282/lh5419sup1.cif

Structure factors: contains datablock(s) I. DOI: 10.1107/S1600536812011282/lh5419Isup2.hkl

Supplementary material file. DOI: 10.1107/S1600536812011282/lh5419Isup3.cml

Additional supplementary materials:  crystallographic information; 3D view; checkCIF report

## Figures and Tables

**Table 1:** Hydrogen-bond geometry (Å, °)

*D*—H⋯*A*	*D*—H	H⋯*A*	*D*⋯*A*	*D*—H⋯*A*
C6—H6*A*⋯O3^i^	0.96	2.64	3.523 (3)	153
C7—H7*C*⋯O3^ii^	0.96	2.61	3.276 (4)	127
C8—H8*A*⋯O3^iii^	0.96	2.64	3.575 (3)	164

Symmetry codes: (i) 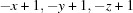; (ii) 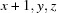; (iii) 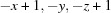.

**Fig. 1.**
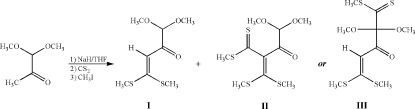
Reaction scheme and possible products.

**Fig. 2.**
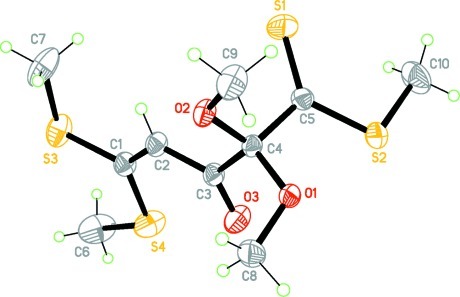
The molecular structure of the title compound, showing 50% probability displacement.

**Fig. 3.**
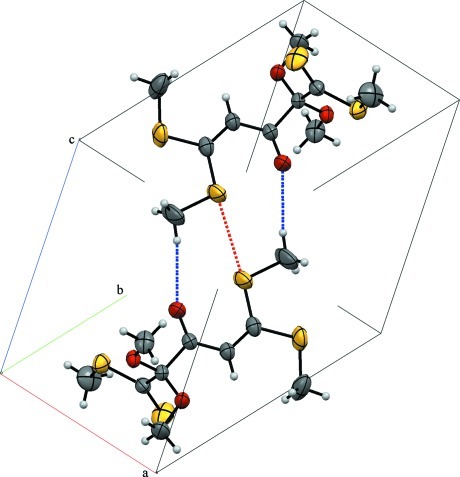
View of the centrosymmetric unit cell, with the short intermolecular S···S contact as red dotted line and C—H···O interactions as blue dashed lines. The rest contacts have been omitted for clarity.

## supplementary crystallographic information

### Comment

In preparation of α-oxoketene dithioactal (I) (Mahata *et al.*, 2003) (Fig. 1), a red-crystalline compound sometimes is derived as a byproduct of the reaction particularly, when NaH, CS_2_ and CH_3_I are used in excess amounts. In order to identify this compound, the red-crystals were investigated using elemental analysis and convenient spectroscopic methods. These investigations show that the byproduct should be a methyl dithioformated derivative of the α-oxoketene dithioactal (I) and it may be one of the two probable structures (II) and (III) (see Fig .1). In order to find out the exact structure of byproduct, the red-prismatic crystals were investigated using single crystal X-ray crystallography.

The molecular structure of the title compound is shown in Fig. 2 and the structure (III) is confirmed as byproduct of the mentioned reaction. In the structure of this compound, the carbonyl group, ethylenic double bond and two connected methylthio groups are coplanar (the torsion angles of C1—C2—C3—O3, S4—C1—C2—C3, C6—S4—C1—C2 and C7—S3—C1—C2 are 0.8 (3)°, -0.4 (3)°, 179.4 (2)° and 1.4 (2)°, respectively). This co-planarity not only, facilitates conjugation of the S lone pairs, the C=C, and the C=O but also, make possible the attractive S···O interaction between S4 and O3 atoms (Ángyán *et al.*, 1987). Due to this attraction the intramolecular S4···O3 non-bonded distance [2.726 (2) Å] is shorter than the sum of the corresponding van der Waals radii (3.25 Å) and the molecule adopts s-*trans*/s-*cis* conformational arrangement for S4—C1 and C2—C3 bonds, respectively (Ángyán *et al.*, 1985; Dixit *et al.*, 1995). Of course, the s-*cis* orientation about the C2—C3 bond minimizes unfavourable steric interaction between S4 and the methyl groups centred on C8 and C9 which is present in the other possible conformation. In this geometry, the arrangement of three atoms C6—S4···O3, like similar part in another molecules (Dixit *et al.*, 1995; Ángyán *et al.*, 1987; Hamilton & LaPlaca, 1964), is almost linear (the measure angle of C6—S4—O3 is 178.5 (1)°).

In the molecule, the bond length of CH_3_—S4 (1.813 (4) Å) is slightly longer than the similar CH_3_—S3 bond (1.784 (3) Å). In addition to a short distance between non-bonded atoms S4 and O3, this observation shows that the intramolecular S···O interaction, might be responsible for lengthening the C6—S4 bond length in the molecule.

In the crystal, molecules are linked into centrosymmetric dimers *via* weak intermolecular C—H···O and S···S interactions [intermolecular S···S distance is 3.405 (3)Å] (Fig. 3). These dimers are linked by further weak C—H···O interactions into columns along the *a* axis.

### Experimental

The title compound was produced as a byproduct of the reaction of synthesis of α-oxoketene dithioactal (I) (Mahata *et al.*, 2003), when NaH, CS_2_ and CH_3_I were used in excess amounts. The melting point of the title compound is 392-394K. The suitable single crystals for X-ray analysis were obtained from ethyl acetate solution at room temperature.

### Refinement

All hydrogen atoms were positioned geometrically with C—H distances = 0.93–0.96 Å and included in a riding model approximation with *U*_iso_(H) = 1.2 or 1.5*U*_eq_(C). A rotating group model was applied to the methyl groups.

### Crystal data

**Table:**

C_10_H_16_O_3_S_4_	*Z* = 2
*M**_r_* = 312.47	*F*(000) = 328
Triclinic, *P*1	*D*_x_ = 1.377 Mg m^−^^3^
Hall symbol: -P 1	Mo *K*α radiation, λ = 0.71069 Å
*a* = 7.114 (5) Å	Cell parameters from 6660 reflections
*b* = 10.404 (5) Å	θ = 1.8–28.5°
*c* = 11.151 (5) Å	µ = 0.62 mm^−^^1^
α = 70.426 (5)°	*T* = 291 K
β = 88.549 (5)°	Block, orange
γ = 76.081 (5)°	0.35 × 0.25 × 0.12 mm
*V* = 753.4 (7) Å^3^	

### Data collection

**Table:**

Stoe IPDS II Image Plate diffractometer	4016 independent reflections
Radiation source: fine-focus sealed tube	2376 reflections with *I* > 2σ(*I*)
Graphite monochromator	*R*_int_ = 0.036
Detector resolution: 0.15 pixels mm^-1^	θ_max_ = 29.2°, θ_min_ = 1.9°
ω scans	*h* = −9→8
Absorption correction: multi-scan [*MULABS* (Blessing, 1995) in *PLATON* (Spek, 2009)]	*k* = −14→11
*T*_min_ = 0.927, *T*_max_ = 1.000	*l* = −15→15
8030 measured reflections	

### Refinement

**Table:**

Refinement on *F*^2^	Secondary atom site location: difference Fourier map
Least-squares matrix: full	Hydrogen site location: inferred from neighbouring sites
*R*[*F*^2^ > 2σ(*F*^2^)] = 0.037	H-atom parameters constrained
*wR*(*F*^2^) = 0.085	*w* = 1/[σ^2^(*F*_o_^2^) + (0.0464*P*)^2^] where *P* = (*F*_o_^2^ + 2*F*_c_^2^)/3
*S* = 0.81	(Δ/σ)_max_ = 0.001
4016 reflections	Δρ_max_ = 0.29 e Å^−^^3^
160 parameters	Δρ_min_ = −0.35 e Å^−^^3^
0 restraints	Extinction correction: *SHELXTL* (Sheldrick, 2008), Fc^*^=kFc[1+0.001xFc^2^λ^3^/sin(2θ)]^-1/4^
Primary atom site location: structure-invariant direct methods	Extinction coefficient: 0.098 (4)

### Special details

**Table:**

Geometry. All e.s.d.'s (except the e.s.d. in the dihedral angle between two l.s. planes) are estimated using the full covariance matrix. The cell e.s.d.'s are taken into account individually in the estimation of e.s.d.'s in distances, angles and torsion angles; correlations between e.s.d.'s in cell parameters are only used when they are defined by crystal symmetry. An approximate (isotropic) treatment of cell e.s.d.'s is used for estimating e.s.d.'s involving l.s. planes.
Refinement. Refinement of *F*^2^ against ALL reflections. The weighted *R*-factor *wR* and goodness of fit *S* are based on *F*^2^, conventional *R*-factors *R* are based on *F*, with *F* set to zero for negative *F*^2^. The threshold expression of *F*^2^ > σ(*F*^2^) is used only for calculating *R*-factors(gt) *etc*. and is not relevant to the choice of reflections for refinement. *R*-factors based on *F*^2^ are statistically about twice as large as those based on *F*, and *R*- factors based on ALL data will be even larger.

### Fractional atomic coordinates and isotropic or equivalent isotropic displacement parameters (Å^2^)

**Table:**

	*x*	*y*	*z*	*U*_iso_*/*U*_eq_	
S1	0.73599 (10)	0.20993 (7)	0.01253 (5)	0.0735 (2)	
S2	0.42779 (7)	0.09542 (6)	0.16747 (5)	0.05140 (15)	
S3	1.01352 (9)	0.53934 (5)	0.25872 (6)	0.06605 (19)	
S4	0.64728 (11)	0.49989 (6)	0.37727 (7)	0.0726 (2)	
O1	0.68814 (17)	−0.01078 (11)	0.36259 (10)	0.0372 (3)	
O2	0.96657 (17)	0.04619 (12)	0.26878 (12)	0.0408 (3)	
O3	0.5813 (2)	0.25685 (14)	0.36536 (15)	0.0587 (4)	
C1	0.8446 (3)	0.43725 (18)	0.29937 (17)	0.0441 (4)	
C2	0.8648 (3)	0.31478 (17)	0.27448 (17)	0.0413 (4)	
H2A	0.9721	0.2856	0.2321	0.050*	
C3	0.7282 (3)	0.22942 (17)	0.31072 (16)	0.0375 (4)	
C4	0.7663 (2)	0.09135 (16)	0.27575 (15)	0.0334 (4)	
C5	0.6516 (3)	0.13253 (17)	0.14738 (16)	0.0389 (4)	
C8	0.7610 (3)	−0.0573 (2)	0.49293 (17)	0.0560 (5)	
H8A	0.6878	−0.1188	0.5459	0.084*	
H8B	0.7489	0.0228	0.5198	0.084*	
H8C	0.8951	−0.1068	0.5003	0.084*	
C10	0.3143 (4)	0.1743 (3)	0.0087 (2)	0.0780 (8)	
H10A	0.1804	0.1711	0.0115	0.117*	
H10B	0.3792	0.1234	−0.0443	0.117*	
H10C	0.3226	0.2705	−0.0256	0.117*	
C9	1.0298 (3)	−0.0851 (2)	0.2456 (2)	0.0613 (6)	
H9A	1.1668	−0.1037	0.2344	0.092*	
H9B	0.9620	−0.0795	0.1699	0.092*	
H9C	1.0029	−0.1597	0.3169	0.092*	
C6	0.6874 (5)	0.6611 (3)	0.3888 (3)	0.0985 (10)	
H6A	0.5826	0.7025	0.4307	0.148*	
H6B	0.6935	0.7258	0.3048	0.148*	
H6C	0.8074	0.6404	0.4371	0.148*	
C7	1.1925 (4)	0.4482 (2)	0.1811 (3)	0.0852 (9)	
H7A	1.2948	0.4960	0.1584	0.128*	
H7B	1.1343	0.4456	0.1055	0.128*	
H7C	1.2449	0.3538	0.2377	0.128*	

### Atomic displacement parameters (Å^2^)

**Table:**

	*U*^11^	*U*^22^	*U*^33^	*U*^12^	*U*^13^	*U*^23^
S1	0.0834 (5)	0.0964 (5)	0.0390 (3)	−0.0415 (4)	0.0117 (3)	−0.0079 (3)
S2	0.0411 (3)	0.0690 (3)	0.0499 (3)	−0.0187 (2)	−0.0013 (2)	−0.0237 (2)
S3	0.0678 (4)	0.0399 (3)	0.0965 (4)	−0.0292 (3)	0.0069 (3)	−0.0199 (3)
S4	0.0997 (5)	0.0515 (3)	0.0917 (4)	−0.0384 (3)	0.0445 (4)	−0.0454 (3)
O1	0.0419 (7)	0.0372 (6)	0.0357 (6)	−0.0189 (5)	0.0029 (5)	−0.0103 (5)
O2	0.0325 (7)	0.0375 (6)	0.0579 (8)	−0.0118 (5)	0.0069 (5)	−0.0215 (5)
O3	0.0575 (9)	0.0548 (8)	0.0845 (10)	−0.0257 (7)	0.0302 (8)	−0.0437 (8)
C1	0.0525 (12)	0.0352 (9)	0.0463 (10)	−0.0178 (8)	0.0009 (8)	−0.0107 (8)
C2	0.0442 (10)	0.0342 (9)	0.0492 (10)	−0.0157 (8)	0.0052 (8)	−0.0148 (8)
C3	0.0400 (10)	0.0349 (8)	0.0426 (9)	−0.0127 (7)	0.0045 (8)	−0.0172 (7)
C4	0.0352 (9)	0.0324 (8)	0.0376 (9)	−0.0145 (7)	0.0062 (7)	−0.0144 (7)
C5	0.0448 (10)	0.0384 (9)	0.0380 (9)	−0.0142 (8)	0.0055 (8)	−0.0162 (7)
C8	0.0655 (14)	0.0600 (12)	0.0385 (10)	−0.0258 (11)	−0.0055 (9)	−0.0037 (9)
C10	0.0661 (16)	0.1023 (19)	0.0638 (15)	−0.0090 (14)	−0.0242 (12)	−0.0321 (14)
C9	0.0508 (13)	0.0486 (11)	0.0964 (17)	−0.0121 (10)	0.0180 (12)	−0.0407 (11)
C6	0.156 (3)	0.0589 (14)	0.117 (2)	−0.0552 (17)	0.061 (2)	−0.0600 (16)
C7	0.0551 (14)	0.0504 (13)	0.144 (3)	−0.0216 (11)	0.0270 (15)	−0.0208 (15)

### Geometric parameters (Å, º)

**Table:**

S1—C5	1.6264 (19)	C4—C5	1.542 (2)
S2—C5	1.719 (2)	C8—H8A	0.9600
S2—C10	1.800 (2)	C8—H8B	0.9600
S3—C1	1.744 (2)	C8—H8C	0.9600
S3—C7	1.784 (3)	C10—H10A	0.9600
S4—C1	1.740 (2)	C10—H10B	0.9600
S4—C6	1.814 (2)	C10—H10C	0.9600
O1—C4	1.3941 (19)	C9—H9A	0.9600
O1—C8	1.436 (2)	C9—H9B	0.9600
O2—C4	1.398 (2)	C9—H9C	0.9600
O2—C9	1.439 (2)	C6—H6A	0.9600
O3—C3	1.219 (2)	C6—H6B	0.9600
C1—C2	1.367 (2)	C6—H6C	0.9600
C2—C3	1.432 (2)	C7—H7A	0.9600
C2—H2A	0.9300	C7—H7B	0.9600
C3—C4	1.572 (2)	C7—H7C	0.9600
			
C5—S2—C10	102.90 (11)	H8A—C8—H8C	109.5
C1—S3—C7	104.16 (11)	H8B—C8—H8C	109.5
C1—S4—C6	104.69 (12)	S2—C10—H10A	109.5
C4—O1—C8	116.18 (13)	S2—C10—H10B	109.5
C4—O2—C9	115.01 (13)	H10A—C10—H10B	109.5
C2—C1—S4	121.38 (15)	S2—C10—H10C	109.5
C2—C1—S3	123.12 (16)	H10A—C10—H10C	109.5
S4—C1—S3	115.49 (11)	H10B—C10—H10C	109.5
C1—C2—C3	122.98 (18)	O2—C9—H9A	109.5
C1—C2—H2A	118.5	O2—C9—H9B	109.5
C3—C2—H2A	118.5	H9A—C9—H9B	109.5
O3—C3—C2	124.82 (16)	O2—C9—H9C	109.5
O3—C3—C4	116.89 (15)	H9A—C9—H9C	109.5
C2—C3—C4	118.28 (16)	H9B—C9—H9C	109.5
O1—C4—O2	112.90 (13)	S4—C6—H6A	109.5
O1—C4—C5	105.88 (13)	S4—C6—H6B	109.5
O2—C4—C5	113.20 (14)	H6A—C6—H6B	109.5
O1—C4—C3	111.57 (14)	S4—C6—H6C	109.5
O2—C4—C3	107.27 (13)	H6A—C6—H6C	109.5
C5—C4—C3	105.87 (13)	H6B—C6—H6C	109.5
C4—C5—S1	121.73 (14)	S3—C7—H7A	109.5
C4—C5—S2	112.04 (12)	S3—C7—H7B	109.5
S1—C5—S2	126.14 (11)	H7A—C7—H7B	109.5
O1—C8—H8A	109.5	S3—C7—H7C	109.5
O1—C8—H8B	109.5	H7A—C7—H7C	109.5
H8A—C8—H8B	109.5	H7B—C7—H7C	109.5
O1—C8—H8C	109.5		
			
C6—S4—C1—C2	179.44 (18)	O3—C3—C4—O1	−30.0 (2)
C6—S4—C1—S3	0.46 (16)	C2—C3—C4—O1	151.22 (14)
C7—S3—C1—C2	1.4 (2)	O3—C3—C4—O2	−154.10 (16)
C7—S3—C1—S4	−179.66 (13)	C2—C3—C4—O2	27.09 (19)
S4—C1—C2—C3	−0.4 (3)	O3—C3—C4—C5	84.76 (19)
S3—C1—C2—C3	178.45 (13)	C2—C3—C4—C5	−94.06 (18)
C1—C2—C3—O3	0.8 (3)	O1—C4—C5—S1	−160.45 (12)
C1—C2—C3—C4	179.49 (16)	O2—C4—C5—S1	−36.26 (19)
C8—O1—C4—O2	60.83 (19)	C3—C4—C5—S1	80.98 (17)
C8—O1—C4—C5	−174.79 (15)	O1—C4—C5—S2	22.77 (16)
C8—O1—C4—C3	−60.07 (19)	O2—C4—C5—S2	146.96 (11)
C9—O2—C4—O1	52.8 (2)	C3—C4—C5—S2	−95.80 (14)
C9—O2—C4—C5	−67.44 (19)	C10—S2—C5—C4	172.39 (14)
C9—O2—C4—C3	176.15 (15)	C10—S2—C5—S1	−4.21 (16)

### Hydrogen-bond geometry (Å, º)

**Table:**

*D*—H···*A*	*D*—H	H···*A*	*D*···*A*	*D*—H···*A*
C6—H6*A*···O3^i^	0.96	2.64	3.523 (3)	153
C7—H7*C*···O3^ii^	0.96	2.61	3.276 (4)	127
C8—H8*A*···O3^iii^	0.96	2.64	3.575 (3)	164

Symmetry codes: (i) −*x*+1, −*y*+1, −*z*+1; (ii) *x*+1, *y*, *z*; (iii) −*x*+1, −*y*, −*z*+1.
